# 2D LiDAR SLAM Back-End Optimization with Control Network Constraint for Mobile Mapping

**DOI:** 10.3390/s18113668

**Published:** 2018-10-29

**Authors:** Jingren Wen, Chuang Qian, Jian Tang, Hui Liu, Wenfang Ye, Xiaoyun Fan

**Affiliations:** GNSS Research Centre, Wuhan University, 129 Luoyu Road, Wuhan 430079, China; jrwen@whu.edu.cn (J.W.); qc_gnss@whu.edu.cn (C.Q.); Loweliu@whu.edu.cn (H.L.); 2013301610119@whu.edu.cn (W.Y.); 2017286180031@whu.edu.cn (X.F.)

**Keywords:** SLAM, Delaunay triangulation network, distance constraint, back-end optimization, mobile mapping

## Abstract

Simultaneous localization and mapping (SLAM) has been investigated in the field of robotics for two decades, as it is considered to be an effective method for solving the positioning and mapping problem in a single framework. In the SLAM community, the Extended Kalman Filter (EKF) based SLAM and particle filter SLAM are the most mature technologies. After years of development, graph-based SLAM is becoming the most promising technology and a lot of progress has been made recently with respect to accuracy and efficiency. No matter which SLAM method is used, loop closure is a vital part for overcoming the accumulated errors. However, in 2D Light Detection and Ranging (LiDAR) SLAM, on one hand, it is relatively difficult to extract distinctive features in LiDAR scans for loop closure detection, as 2D LiDAR scans encode much less information than images; on the other hand, there is also some special mapping scenery, where no loop closure exists. Thereby, in this paper, instead of loop closure detection, we first propose the method to introduce extra control network constraint (CNC) to the back-end optimization of graph-based SLAM, by aligning the LiDAR scan center with the control vertex of the presurveyed control network to optimize all the poses of scans and submaps. Field tests were carried out in a typical urban Global Navigation Satellite System (GNSS) weak outdoor area. The results prove that the position Root Mean Square (RMS) error of the selected key points is 0.3614 m, evaluated with a reference map produced by Terrestrial Laser Scanner (TLS). Mapping accuracy is significantly improved, compared to the mapping RMS of 1.6462 m without control network constraint. Adding distance constraints of the control network to the back-end optimization is an effective and practical method to solve the drift accumulation of LiDAR front-end scan matching.

## 1. Introduction

With the rapid development of the geospatial information service industry, the demand for geospatial data is also growing enormously. The mobile mapping system has also been rapidly developing in recent years due to its ability to provide efficient, fast, and complete data collection functions. Simultaneous localization and mapping (SLAM) is considered to be an effective method for solving positioning and mapping problems in GNSS-denied areas, as it unifies positioning and mapping problems in a single framework, which has been intensively investigated in the robotics community for two decades [1]. SLAM builds a consistent map of the environment incrementally and determines its location within this map simultaneously [2]. SLAM is essentially a probability-based optimal estimation problem. One advantage of the probability estimation algorithm is that it can stably measure the noise in the environment and indicate the uncertainty in the measurement and estimation process [3].

There are three basic SLAM paradigms, from which most others are derived. The first, known as Extended Kalman Filter (EKF) based SLAM, is the earliest and probably the most influential algorithm. There are various forms of EKF based algorithms that have been proposed for a variety of different tasks and environments; the EKF based SLAM method has been studied in depth [4,5]. EKF based SLAM can achieve better results when the sensor noise satisfies the Gaussian distribution assumption and the system nonlinearity is small [6]. A key concern of the EKF based approach to SLAM lies in the quadratic nature of the covariance matrix: as the landmark points continue to increase, the covariance matrix of the system increases in a quadratic manner, resulting in a large computational pressure [7]. EKF based SLAM is not suitable for large-scale environments. The second SLAM paradigm is based on particle filters. Particle filter is also a kind of Bayesian estimation which represents a posterior probability through a set of particles. The core idea in particle filter is to express their distribution by taking examples of random states extracted from posterior probabilities [7]. The advantage of particle filter over EKF is that, instead of model linearization, it uses some samples to obtain the state estimation. Particle filter has the ability to handle nonlinear, non-Gaussian distribution, and multimodal problems [8]. Rao–Blackwellized particle filtering (RBPF), derived from particle filter, was thus applied in the algorithm proposed by Montemerlo, named FastSLAM [9]. However, the number of particles required for particle filter increases exponentially with the underlying state space (the space of maps and robot position) [7]. The more complicated the environment is, the more particles are required, and the higher the complexity of the algorithm. In addition, the resampling phase can result in loss of sample validity and diversity, leading to sample depletion. How to maintain the validity and diversity of particles and overcome the sample depletion is another research focus of particle filter [3]. Due to the linearization and updating efficiency of the filter-based SLAM method, it is not applicable to large-scale environments. The third SLAM paradigm is graph-based SLAM, which solves the SLAM problem through nonlinear sparse optimization [7]. Graph-based SLAM draws intuition from the fact that the SLAM problem can be modeled as a sparse graph of constraints, where the nodes represent the state of the system composed of the robot and the environment at different times, and the edges represent the spatial constraints between the nodes [10]. The constraints generally arise from scan matching and observations, such as from an odometer, Inertial Measurement Unit (IMU), and so on. Graph-based SLAM is usually divided into the front-end and back-end. The front-end constructs the nodes and edges of the graph according to the observation values and system constraints. The back-end applies optimization techniques to complete the graph optimization. Since the graph-based SLAM method utilizes all the observations information to optimize the robot’s complete trajectory and environment, we can get a globally consistent trajectory and map, so it is also known as the full SLAM method [10]. The graph-based SLAM method has the advantage that it can scale to much higher-dimensional maps than EKF based SLAM, the update time of the graph is constant, and the amount of memory required is linear (under some mild assumptions) [11]. It is also easy for graph-based SLAM methods to handle the data association problems, which are usually addressed by the SLAM front-end, because it is easy to integrate additional knowledge (constraints) into data association. Compared to EKF based SLAM and particle filter based SLAM methods, graph-based SLAM has undergone a renaissance and currently belongs among the state-of-the-art techniques with respect to speed and accuracy [10].

In the framework of graph-based SLAM, the front-end is responsible for providing the initial values of the location and map, and the back-end is responsible for optimizing all the poses of scans and submaps. However, due to the existence of observation errors and data association errors, the errors generated previously will inevitably accumulate to the next moment, which will cause incorrect location and mapping results. In order to eliminate the cumulative errors and build globally consistent tracks and maps, various methods have already been addressed in previous works. A nature idea in SLAM is recognizing previously visited places to reduce cumulative errors, a process known as loop closure detection. Loop closure detection refers to the robot recognizing the place where it has been, thus establishing the relationship between the current data and all previous data, and then adding new constraints to the back-end to eliminate error accumulation. Popular loop closure detection approaches can be divided into two categories: the appearance-based approach and the optimization-based approach [12]. The underlying idea behind the appearance-based approach is that loop closure detection is done by comparing all previous “images” with the new one [13]. The core issue in the appearance-based approach is how to calculate similarity between previous “images” and the new one. Generally, we extract distinctive features and describe them in a mathematical language, where the features are known as descriptors, and then we can calculate the similarity score between “images” by the descriptors [14,15,16,17]. Deep learning and machine learning can also be employed for loop closure detection [12,18]. The appearance-based approach is widely used in the computer vision community, and many mature algorithms have been proposed, as an image contains abundant information that can be used to extract distinctive features. However, compared to images, 2D LiDAR scans encode much less information (such as intensity gradients and some structured features, e.g., corners have weak variations in the range measurements), so it is difficult to extract distinctive features in 2D (LiDAR) scans for loop closure detection [12]. The optimization-based approach is represented by Google’s Cartographer LiDAR SLAM algorithm, which combines scan-to-submap matching with loop closure detection and graph optimization. The key idea in Cartographer is to build multiple submaps and align new scans to nearby submaps to generate constraints on a graph. In order to achieve real-time loop closure, a branch-and-bound approach for computing scan-to-submap matches as constraints is used in Cartographer [19]. All loop closure detection approaches and the optimization-based approach of Cartographer are based on the fact the robot will go back to where it has already been; however, in some special mobile mapping scenarios, there are no loop closure conditions, such as in a U-shaped mapping scene, where the robot cannot go back to where it has already been. Another way is to add additional sensors to assist SLAM, such as GNSS, INS, and so on. In [20,21], GNSS/INS is utilized to improve the positioning accuracy of the SLAM result. Unlike SLAM techniques, GNSS sensors do not drift over time; however, GNSS signals are not always available and suffer from signal occlusion and severe multipath effects in urban or indoor environments. At present, combining additional sensors increases the complexity and hardware cost of the SLAM system. SLAM problems focus on detecting the environment without taking any special prior knowledge into account; however, prior knowledge can improve the accuracy of the SLAM result greatly, as prior knowledge can be added to the SLAM problem as constraints to reduce the cumulative error. The use of prior knowledge in graph-based SLAM has been exploited by experts and scholars. Kümmerle et al. have used the correspondences detected between 3D scans and publicly available aerial images as prior knowledge to achieve global consistency [22], and Schuster et al. use a landmark map as prior knowledge to improve location accuracy based on GraphSLAM [23]. However, this method also needs to extract feature points and find constraint relationships by feature point matching.

Although the use of prior knowledge in SLAM has been studied before, different from other prior knowledge, distance measurement is an easy-to-measure and easy-to-use additional observation, and a stable geometry can be formed between the distance measurements to effectively eliminate cumulative errors when there are three or more distance measurements. In this paper, we first propose the method of adding distance constraints of control network to the back-end optimization to effectively eliminate cumulative errors, we analyzed the necessary conditions for the distance constraints of control network to work and analyzed the effect of different control network construction methods on the slam mapping results. The field test shows that the algorithm proposed is an effective way to eliminate cumulative errors and improve the accuracy of the SLAM mapping result obviously when there are no loop closure conditions in large-scale GNSS-dined area, for example in a narrow corridor or a U shaped area or in a large parking lot.

The remainder of this paper is organized as follows: Section 2 gives the detailed parameters of the hardware; Section 3 describes the methods used in this paper; and Section 4 introduces the experimental results and draws the conclusion.

## 2. System Overview

**Figure 1:**
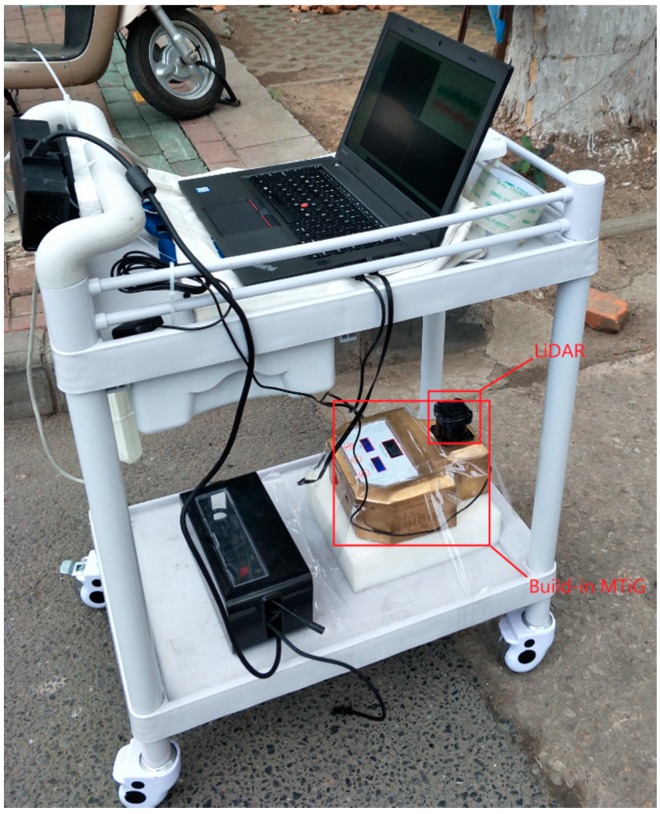
System hardware platform.

In order to verify the performance of the algorithm described in the following section, a LiDAR/IMU integrated system (Figure 1) was designed and implemented. The measurement sensor LiDAR and inertial measurement unit (IMU) are integrated on the mobile mapping platform to form the hardware of the system. A “UTM-30LX-EW” LiDAR system, which was manufactured by the Hokuyo Company (Osaka, Japan), was adopted for the platform. The LiDAR system operates at 40 Hz, has a scanning angle range of 270° with an angular resolution of 0.25°, and has a maximum effective range of 30 m with a range accuracy of ±30 mm at 0.1 m–10 m, and ±50 mm at 10 m–30 m. The measurement accuracy of the LiDAR system is in millimeters and it is a medium-precision device. The model of IMU is MTiG, the bias stability of the IMU gyroscopes is about 200.0°/h, the bias stability of the IMU accelerators is about 2000 mGal (1 Gal = 1 cm/s), and the sampling frequency of the IMU is 200 Hz. According to the technical indicators, the IMU we adopted is a MEMS-level (Micro-Electro-Mechanical System) device.

In order to get the distance constraints, we firstly need to layout survey control network and use a total station to survey in the area to be mapped. Here, a TIANYU CST-632 total station (GuangZhou, China) was adopted. The angle accuracy of the total station was 2 s and the range accuracy was $\pm (2+2\times 10^{-6}*D)$ mm.

Finally, to measure the mapping accuracy of the proposed algorithm, a high-precision terrestrial laser scanner, FARO Focus3D X130 HDR (Florida, USA), was used to map the experimental area with millimeter accuracy. The detailed parameters of all hardware devices are shown in Table 1.

## 3. Method

**Figure 2:**
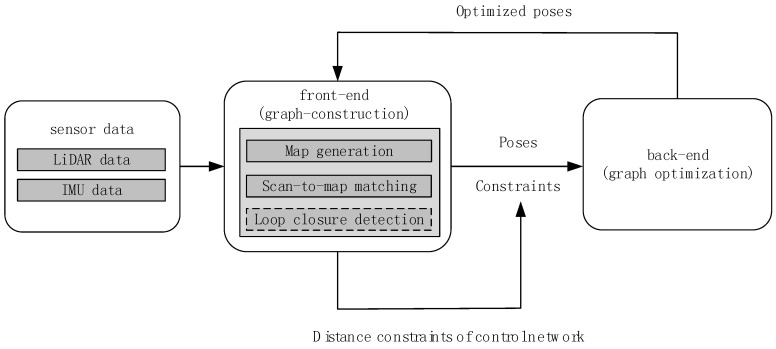
Graph-based SLAM framework.

The framework of the graph-based SLAM used in this paper is shown in Figure 2. The framework is divided into the front-end and back-end. The front-end mainly completes map generation and scan-to-map matching to provide the initial poses, and it is also responsible for loop closure detection to provide constraints for the back-end. The back-end mainly completes graph optimization to optimize all the poses. While in this paper we consider the case where there are no loop closure conditions, we propose the addition of extra distance constraints of the control network to the back-end optimization. This method requires one to prearrange and measure the control network in the survey area. We align the LiDAR scan center with the control point of the presurveyed control network and mark the scanning frame to obtain the distance constraint between the marked scan frames.

### 3.1. Multiresolution Map Generation

The occupancy grid map is a location-based representation of the environment, which has become a dominant paradigm for environment modeling in mobile robotics in the past two decades. The environment will be divided into a series of uniform grids, and the occupancy probability, which indicates the probability that each cell is occupied, is estimated based on sensor measurements [24]. An occupancy grid map was used in this paper to represent the environment, as occupancy grid maps are intuitive, easy to expand and maintain, and are especially suitable for processing laser and ultrasonic data. Multiresolution occupancy grid maps are similar to image pyramids in computer vision: the map is initialized with the total number of map layers, the minimum resolution of the map, the center of the map, and the geographic boundary value. The map is divided from top to bottom until it is divided into a grid of the smallest resolution. When searching for a map, the matching search is performed from coarse to fine, and the lower resolution layer is matched with lower precision, then is further searched on a finer layer, resulting in higher precision results. Different levels of maps are stored in the memory, and they are updated synchronously by the pose estimation results obtained during the matching process, which ensures that maps with different resolutions are consistent at the same time. Moreover, avoiding the problem of increased computation due to operations such as downsampling, and the storing of relative poses in the memory is very convenient for the back-end optimization.

**Figure 3:**
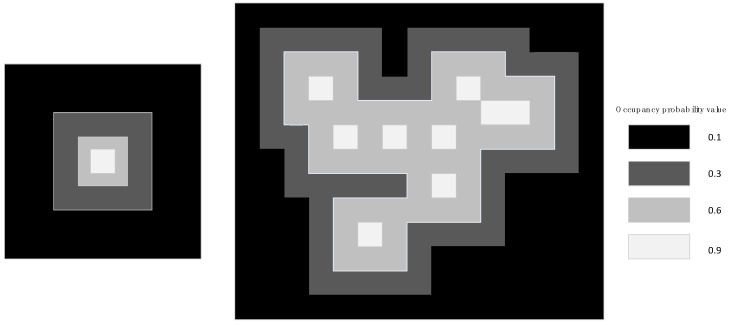
Probability representation of occupancy grid map.

The assignment of the occupancy grid map strategy is inspired by the previous work of Tang [25]. Every grid in the occupancy grid map as shown in Figure 3 and each grid is generally given by any predefined occupancy probability values, e.g., 0.1, 0.3, 0.6, and 0.9, which represent the occupancy probability that an obstacle exists in the grid. All grids in the map will be initialized with the occupancy probability value of 0.1. If any grid cell is occupied by a LiDAR scan point when a new LiDAR scan is projected onto the occupancy grid map, the occupancy probability will be set to 0.9, and the occupancy probability values of the two circles of the grid around this grid will be set to 0.6 and 0.3. The occupancy probability value will be set to the larger one if it is repeatedly assigned.

### 3.2. Front-End Scan-to-Map Matching

Scan matching is a process of relative positioning, which utilizes two or more consecutive frames of scan points to calculate the pose of the moving platform. The scan-matching algorithm has been proved to be one of the most frequently relied-upon algorithms for determining the pose of mobile platforms in the SLAM community. There are two main types of scan-matching algorithms: scan-to-scan matching and scan-to-map matching. The classic scan-to-scan matching algorithms are iterative closed point (ICP) [26] and iterative closed line (ICL) [27,28], and they calculate a relative pose by two scans adjacent to each other. The main disadvantage of many ICP-based and ICL-based methods is the expensive search for point or line correspondences, which has to be done in every iteration. It is also obvious that these methods will quickly accumulate errors as they only use a small amount of observation information. The scan-to-map matching algorithm involves not only the matching between the two scans adjacent to each other, but also the matching between the current scan and the existing map, which can obtain a more accurate result and help limit the accumulation of errors. Kohlbrecher implemented a scan-to-map algorithm based on the Gauss–Newton approach [29]. The basic idea in this scan-to-map matching algorithm is using a Gauss–Newton approach to find a local optimal solution to align the LiDAR scans with the map learnt so far, which was often used in computer vision. Scan-to-map matching is efficient and robust when the initial pose is given correctly, and the initial pose is usually given by IMU.

Given an occupancy grid map constructed from the previous scans and an initial pose of a scan, the Gauss–Newton algorithm is iterated to seek a rigid transformation that minimizes Equation (1):

(1) $$\xi^{*}=\underset{\xi }{\arg\min}\sum_{i=1}^{n}{\left[1-M(S_{i}(\xi ))\right]}^{2}.$$

That is, we want to find the rigid transformation that maximizes the probabilities at the scan points in the map. Here, x,y,ψ indicates the position and heading angle of the mobile carrier in the world coordinate system; n indicates the number of scan points; and $S_{i}(\xi )$ is a function of ξ (Equation (2)), which converts a LiDAR scan point $s_{i}=(s_{i,x},s_{i,y})$ to the world coordinate system:

(2) $$s_{i}(\xi )=\left(\begin{array}{cc} \cos(\psi ) & -\sin(\psi )\\ \sin(\psi ) & \cos(\psi ) \end{array}\right)\left(\begin{array}{c} s_{i,x}\\ s_{i,y} \end{array}\right)+\left(\begin{array}{c} x\\ y \end{array}\right).$$

$M(S_{i}(\xi ))$ will return the occupancy probability at the coordinates given by $S_{i}(\xi )$. The Gauss–Newton iterative algorithm is a gradient descent method; however, as the discrete nature of occupancy grid maps does not allow the direct computation of interpolated values or gradients, the method of bilinear interpolation is adopted to calculate the occupancy probability and gradient. Intuitively, the grid map cell values can be viewed as samples of an underlying continuous probability distribution [26]. For a point $P_{m}$ in a continuous map, the occupancy probability $M(P_{m})$ and gradient $\nabla M(P_{m})=(\frac{\partial M}{\partial x}(P_{m}),\frac{\partial M}{\partial y}(P_{m}))$ can be approximated by bilinear interpolation using the nearest four integer coordinates around it. A reasonable initial pose is very important for the Gauss–Newton iterative algorithm, as it determines the iteration number and convergence of the Gauss–Newton algorithm; the initial pose is given by the IMU in this paper.

### 3.3. Back-End Optimization

As a map contains a few dozen recent scans, which are also known as submaps, the cumulative error caused by the scan-to-map matching approach described above is very small. However, as many submaps are created to represent the large-scale environment, the total cumulative error between the submaps will finally corrupt the mapping result of SLAM; thus, it is very important to properly optimize the poses of all scans and all submaps. A submap will be added to loop closure detection when there is no longer a new scan insert. Like scan-to-map matching, the back-end optimization problem can also be formulated as a nonlinear least squares problem which can be expressed as:

(3) $$\begin{array}{c} \arg\min\\ {\left\{M\right\}}_{m},{\left\{S\right\}}_{n} \end{array}\frac{1}{2}\sum_{ij}\rho (E^{2}(\xi_{i}^{m},\xi_{j}^{S};\Sigma_{ij},\xi_{ij})).$$

In this formula, ${\left\{M\right\}}_{m}=\xi_{1}^{M},\xi_{2}^{M}\cdots \xi_{m}^{M}$ represents the poses of all submaps, m; ${\left\{S\right\}}_{n}=\xi_{1}^{S},\xi_{2}^{S}\cdots \xi_{n}^{S}$ represents the poses of all scans, n; $\xi_{ij}$ represents the relative pose between submap i and scan j; $\Sigma_{ij}$ is the covariance matrix which can be obtained from Equation (1); and ρ is a loss function which is used to reduce the effect of outliers that may cause the adding of incorrect scan matching constraints to the back-end optimization problem.

The residuals can be calculated as:

(4) $$E^{2}(\xi_{i}^{m},\xi_{j}^{S};\Sigma_{ij},\xi_{ij})=e(\xi_{i}^{m},\xi_{j}^{S};\xi_{ij})\Sigma_{ij}^{-1}e(\xi_{i}^{m},\xi_{j}^{S};\xi_{ij}),$$

(5) $$e(\xi_{i}^{m},\xi_{j}^{S};\xi_{ij})=\xi_{ij}-\left(\begin{array}{c} R_{\xi_{i}^{m}}^{-1}(t_{\xi_{i}^{m}}-t_{\xi_{j}^{S}})\\ \xi_{i;\theta }^{m}-\xi_{j;\theta }^{S} \end{array}\right).$$

For the back-end optimization problem, it is vital to add correct loop closure constraints to the back-end optimization problem in Equation (3), which can be solved by loop closure detection. When a new scan is added to the map, an estimated pose will be given by the scan-to-match approach, and loop closure detection will be performed by looking for the best possible matches within a search window W in the vicinity of the estimated pose. If a good enough match is found, which can be expressed as:

(6) $$\xi^{*}=\underset{\xi =W}{\arg\min}\sum_{k=1}^{K}M_{nearest}(T_{\xi }T_{k}),$$

where $M_{nearest}$ is the nearest grid point where the grid occupancy probability values are rounded when M is matched with all submaps; then, the loop closure constraint will be added to the back-end optimization to optimize all the poses of scans and submaps. Equation (6) is an optimization problem, and as with all other optimization problems, it has a finite but very large number of feasible solutions and is NP-hard (Non-deterministic Polynomial). Branch-and-bound is an effective and the most widely used tool for solving large-scale NP-hard combinatorial optimization problems [30]. We solve Equation (6) by using the branch-and-bound approach and this is inspired by the work of Cartographer [19].

### 3.4. Back-End Optimization with Distance Constraints of Control Network

**Figure 4:**
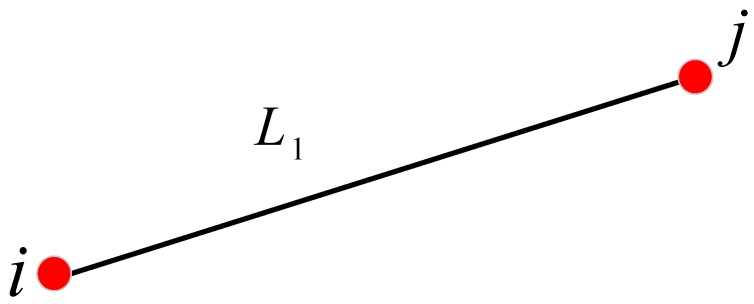
Two marked scans.

If loop closures are correctly detected and added to the back-end optimization in the previous steps, all the poses of scans and submaps will be optimized and cumulative errors will be reduced. Unfortunately, we are not always able to find the loop closure accurately; the loop closure may not even exist at all in some special mapping situations. Under these circumstances, we propose to add distance constraints of the control network to the back-end optimization by aligning the LiDAR scan center with the control point of the presurveyed control network. The Delaunay triangulation is a collection of connected but nonoverlapping triangles, and the circumscribed circles of these triangles do not contain any other points of this region. The Delaunay triangulation has the advantages of good structure, simple data structure, small data redundancy, and high storage efficiency, and thus we used the Delaunay triangulation network theory to generate the control network in this paper. Firstly, according to the conditions of the area to be mapped, a control network is reasonably arranged and surveyed with a total station, and thus the distance between the control points is known. When the LiDAR scanning center of the mobile mapping platform passes over the control point (that is the origin of the carrier coordinate system coincides with the control point), the scanning frame is marked by the data collection software. We can obtain the relative distance between the marked scans, which will be added to the back-end optimization constraint equation to reduce the cumulative error. Taking the two marked scans (Figure 4) as an example, the relative distance between the two marked scans i, j is $L_{1}$ and the current pose in the world is $\left(X_{i}^{0},Y_{i}^{0},\psi_{i}),(X_{j}^{0},Y_{j}^{0},\psi_{j}\right)$.

After optimization, the pose of i, j is 

(7) $${\hat{X}}_{i}=X_{i}^{0}+{\hat{x}}_{i},{\hat{Y}}_{i}^{0}=Y_{i}^{0}+{\hat{y}}_{i}.$$

The residual for the distance constraints can be calculated as:

(8) $$v_{L1}=\sqrt{{\left({\hat{X}}_{i}-{\hat{X}}_{j}\right)}^{2}+{\left({\hat{Y}}_{i}-{\hat{Y}}_{j}\right)}^{2}}-L_{1}.$$

Expanded according to the Taylor series, the residual can be formulated as:

(9) $$v_{L1}=-\frac{\Delta X_{ij}^{0}}{S_{ij}^{0}}{\hat{x}}_{j}-\frac{\Delta Y_{ij}^{0}}{S_{ij}^{0}}{\hat{y}}_{j}+\frac{\Delta X_{ij}^{0}}{S_{ij}^{0}}{\hat{x}}_{i}+\frac{\Delta Y_{ij}^{0}}{S_{ij}^{0}}{\hat{y}}_{i}-(L_{1}-S_{ij}^{0}).$$

In Equation (9),

(10) $$\Delta X_{ij}^{0}=X_{i}^{0}-X_{j}^{0},\Delta Y_{ij}^{0}=Y_{i}^{0}-Y_{j}^{0},S_{ij}^{0}=\sqrt{{\left(X_{i}^{0}-X_{j}^{0}\right)}^{2}+{\left(Y_{i}^{0}-Y_{j}^{0}\right)}^{2}}.$$

By adding *n* distance constraints as in Equation (9) to the back-end optimization in Equation (3), the cumulative errors of distance and angle will be reduced; the result of this will be discussed in the next section.

## 4. Field Test and Results

**Figure 5:**
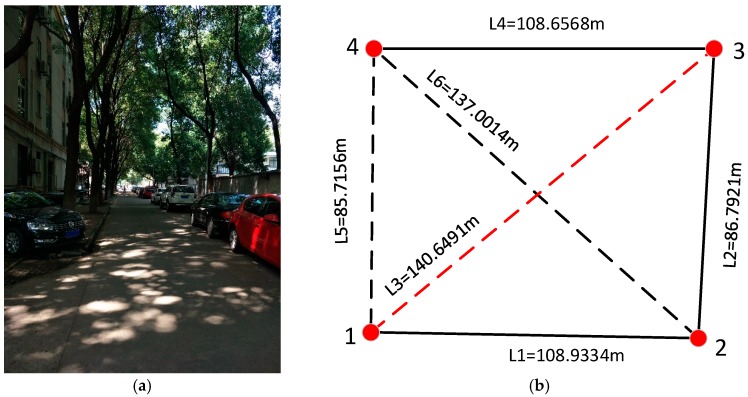
(**a**) field test environment; (**b**) control network.

In order to verify the performance of the algorithm described above, we conducted corresponding field tests. They were carried out in a typical urban GNSS-weak outdoor area in a campus, as shown in Figure 5a, and the survey area was roughly shaped as a “U”-type area. According to the conditions of the area to be mapped, a surveying control network (Figure 5b) containing four control points was reasonably arranged and surveyed with a TIANYU CST-632 total station. The solid line in black in Figure 5b indicates the measurement route. The total length of the measurement route is about 300 m. The LiDAR/IMU integrated system was mounted on a vehicle; when the vehicle was running near the control point, we aligned the LiDAR scan center with the control point (the origin of the carrier coordinate coincides with the control point) by carefully adjusting the movement of the vehicle, and then we marked the scanning frame by the data collection software. The relative distance between two marked scans will be added as constraints to the back-end optimization.

**Figure 6:**
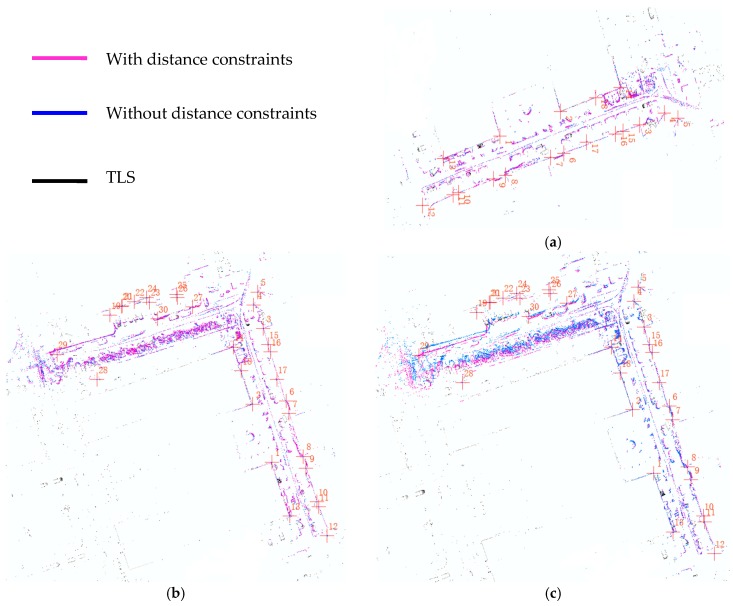
(**a**) result of adding one distance constraint (L1) and result without distance constraint; (**b**) result of adding two distance constraints (L1, L2) and result without distance constraint; (**c**) result of adding three distance constraints (L1, L2, L3) and result without distance constraint.

The corners and walls of the building and other obvious fixed objects are selected as the main feature points for accuracy evaluation, and the high-precision map which was produced by the terrestrial laser scanner FARO Focus3D X130 HDR is used as the reference map. The result map produced by our algorithm and the reference map can be both opened by ArcGIS, we manually extract the positions of the common feature points from the reference map and our result map respectively in ArcGIS. Firstly, we consider the simple case: we separately test the case of adding one constraint edge, two constraint edges, and three constraint edges. The mapping results are shown in Figure 6 and the RMS errors of the coordinates of the selected common feature points are shown in Table 2. From Figure 6, we can intuitively see that the mapping results of adding one or two constraint edges are basically coincident with the mapping result without distance constraint, while the mapping result of adding three constraint edges is obviously improved compared to the mapping result without distance constraints; the RMS values in Table 2 also illustrates this phenomenon. The RMS values of adding one distance constraint (L1) and without the distance constraint are 0.1612 m and 0.1904 m, respectively; and the RMS values of adding two distance constraints (L1, L2) and without the distance constraints are 0.6608 m and 0.7303 m, respectively. Considering the errors brought by manual operation, such as errors caused by manually selecting the common feature point, the mapping results of adding one or two distance constraints and without distance constraints are basically coincident. When adding three distance constraints (L1, L2, L3), the RMS is significantly reduced to 0.2369 m.

The results show that adding one or two distance constraints to the back-end optimization is basically not effective in improving the accuracy of the SLAM results. As having one or two distance constraints is not stable, they are not effective in reducing the angular drift of SLAM. The third test shows that it is effective to improve the accuracy of the SLAM results when there are three distance constraint edges and they can form a triangle, it means that the relative position of the control points are known in the global coordinate system, which is usually determined by the coordinate system of the first scan data in SLAM, as we all know that the triangle is the most stable geometry. The stable triangular structure between the constrained edges can effectively reduce the drift of angle and distance.

**Figure 7:**
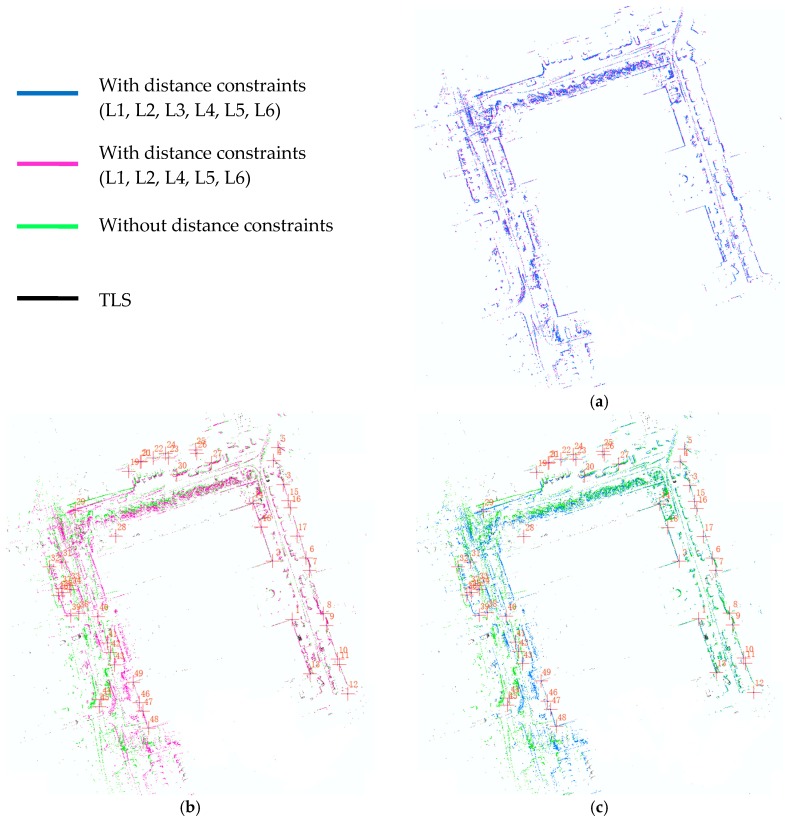
MAP_A: result of adding all distance constraints (L1, L2, L3, L4, L5, L6); MAP_B: result of adding distance constraints of Delaunay triangulation network (L1, L2, L4, L5, L6); MAP_C: result without any distance constraints; MAP_D: high precision TLS reference map; (**a**) comparison of MAP_A and MAP_B; (**b**) comparison of MAP_B and MAP_C, MAP_D; (**c**) comparison of MAP_A and MAP_C, MAP_D.

The above experiments confirm that the distance constraints without stable geometry cannot improve the accuracy of SLAM effectively. Only when different distance constraints (at least three) form a stable triangulation is it effective to reduce the angular and distance drift of SLAM by adding distance constraints to the back-end optimization. According to the number of the points in the control network, there are six distances in the control network, and, according to the Delaunay triangulation network theory, L1, L2, L4, L5, and L6 can form a stable Delaunay triangulation network. The SLAM result of adding all the existing six distance constraints (MAP_A) and the SLAM result of adding the distance constraints of Delaunay triangulation network (MAP_B) are shown in Figure 7 and the RMS errors of the coordinates of the selected common feature points are shown in Table 3. From Figure 7a, we can intuitively see that MAP_A and MAP_B are basically coincident. Compared to the high-precision reference map produced by terrestrial laser scanner, the RMS values of 49 selected common feature points are, respectively, 0.3356 m (MAP_A) and 0.3614 m (MAP_B), while the RMS values in the absence of any distance constraints (MAP_C) is 1.646 m. Considering the error brought by selecting the common feature points, adding all the existing distance constraints or adding the distance constraints of the Delaunay triangulation network basically has the same effect on improving the accuracy of the SLAM result.

## 5. Conclusions

In this paper, a back-end optimization approach with distance constraints of control network in graph-based 2D LiDAR SLAM is first proposed. We align the LiDAR center with the control point and mark the epoch to get the relative distance between the marked epochs. The experimental results show that the distance constraint can suppress the accumulation of positioning errors effectively only if the distance constraints form a stable geometry such as a triangle. Therefore, we connect the control points with the Delaunay triangulation and measure the side lengths as the distance constraints. Moreover, the results show that Delaunay triangulation has similar performance with connecting each control point to each other one. Overall, under the condition of no loop closure, the mapping results of our field tests showed that adding distance constraints of the Delaunay triangulation network to the back-end optimization is an effective way to eliminate cumulative errors and improve the accuracy of the SLAM result.

## Figures and Tables

**Table 1:** The detailed parameters of hardware.

	LiDAR	IMU	
**Product model**	Hokuyo UTM-EX	**Product model**	MEMS-level MTi-G
**Sampling frequency**	10 Hz	**Sample frequency**	200 Hz
**Scan range**	0.1–30 m	**Gyroscope bias**	200°/h
**Scan angle**	270°	**Accelerometer**	2000 mGal (1 Gal = 1 cm/s^2^)
**Angular resolution**	0.25°		
	**Total Station**	**Product model**	
**Product model**	TIANYU CST-632	**Product model**	FARO Focus3D X130 HDR
**Angle Accuracy**	2 s	**Range Accuracy**	±2 mm
**Range Accuracy**	±(2 + 2 × 10^−6^ * D) mm	**Scan range**	0.6–130 m

**Table 2:** RMS of adding different numbers of distance constraints.

Number of Constraints	Track Length	Number of Common Feature Points	RMS (without Constraint)	RMS (with Constraint)
1 (L1)	108.9 m	18	0.1904 m	0.1612 m
2 (L1, L2)	195.6 m	30	0.7303 m	0.6608 m
3 (L1, L2, L3)	195.6 m	30	0.7303 m	0.2369 m

**Table 3:** RMS of adding all existing distance constraints and RMS of adding distance constraints of Delaunay triangulation network.

Number of Constraints	Track Length	Number of Common Feature Points	RMS (without Constraint)	RMS (with Constraint)
6 (L1, L2, L3, L4, L5, L6)	304.3 m	49	1.6462 m	0.3356 m
5 (L1, L2, L4, L5, L6)	304.3 m	49	1.6462 m	0.3614 m

## References

[1] Bailey T., Durrant-Whyte H. (2006). Simultaneous localization and mapping (SLAM): Part II. IEEE Robot. Autom. Mag..

[2] Hartley R., Zisserman A. (2000). Multiple View Geometry in Computer Vision.

[3] Thrun S., Burgard W., Fox D. (2005). Probabilistic Robotics.

[4] Xie X., Yu Y., Lin X., Sun C. An EKF SLAM algorithm for mobile robot with sensor bias estimation. Proceedings of the 2017 32nd Youth Academic Annual Conference of Chinese Association of Automation.

[5] Zhang T., Wu K., Song J., Huang S., Dissanayake G. (2017). Convergence and Consistency Analysis for A 3D Invariant-EKF SLAM. IEEE Robot. Autom. Lett..

[6] Huang S., Dissanayake G. (2007). Convergence and Consistency Analysis for Extended Kalman Filter Based SLAM. IEEE Trans. Robot..

[7] Thrun S., Leonard J.J. (2015). Simultaneous Localization and Mapping. IEEE Trans. Intell. Transp. Syst..

[8] Yatim N.M., Buniyamin N. (2015). Particle filter in simultaneous localization and mapping (Slam) using differential drive mobile robot. J. Teknol..

[9] Montemerlo M.S. (2003). Fastslam: A Factored Solution to the Simultaneous Localization and Mapping Problem with Unknown Data Association. Ph.D. Thesis.

[10] Grisetti G., Kummerle R., Stachniss C., Burgard W. (2010). A tutorial on graph-based SLAM. IEEE Intell. Transp. Syst. Mag..

[11] Thrun S. (2007). Simultaneous localization and mapping. Robotics and Cognitive Approaches to Spatial Mapping.

[12] Li J., Zhan H., Chen B.M., Reid I., Lee G.H. Deep learning for 2D scan matching and loop closure. Proceedings of the 2017 IEEE/RSJ International Conference on Intelligent Robots and Systems (IROS).

[13] Labbé M., Michaud F. Online global loop closure detection for large-scale multi-session graph-based SLAM. Proceedings of the 2014 IEEE/RSJ International Conference on Intelligent Robots and Systems.

[14] Magnusson M., Andreasson H., Nuchter A., Lilienthal A.J. (2010). Appearance-based loop detection from 3D laser data using the normal distributions transform. J. Field Robot..

[15] Bosse M., Zlot R. (2009). Keypoint design and evaluation for place recognition in 2D lidar maps. Robot. Auton. Syst..

[16] Tipaldi G.D., Spinello L., Burgard W. Geometrical FLIRT phrases for large scale place recognition in 2D range data. Proceedings of the 2013 IEEE International Conference on Robotics and Automation.

[17] Himstedt M., Frost J., Hellbach S., Böhme H.-J., Maehle E. Large scale place recognition in 2D LIDAR scans using Geometrical Landmark Relations. Proceedings of the 2014 IEEE/RSJ International Conference on Intelligent Robots and Systems.

[18] Callmer J., Ramos F., Nieto J. Learning to detect loop closure from range data. Proceedings of the IEEE International Conference on Robotics and Automation.

[19] Hess W., Kohler D., Rapp H., Andor D. Real-time loop closure in 2D LIDAR SLAM. Proceedings of the IEEE International Conference on Robotics and Automation.

[20] Qian C., Liu H., Tang J., Chen Y., Kaartinen H., Kukko A., Zhu L., Liang X., Chen L., Hyyppä J. (2016). An Integrated GNSS/INS/LiDAR-SLAM Positioning Method for Highly Accurate Forest Stem Mapping. Remote Sens..

[21] Hening S., Ippolito C.A., Krishnakumar K.S., Stepanyan V., Teodorescu M. 3D LiDAR SLAM Integration with GPS/INS for UAVs in Urban GPS-Degraded Environments. Proceedings of the AIAA Information Systems-AIAA Infotech @ Aerospace.

[22] Kümmerle R., Steder B., Dornhege C., Kleiner A., Grisetti G., Burgard W. (2011). Large scale graph-based SLAM using aerial images as prior information. Auton. Robot..

[23] Schuster F., Keller C.G., Rapp M., Haueis M., Curio C. Landmark based radar SLAM using graph optimization. Proceedings of the IEEE International Conference on Intelligent Transportation Systems.

[24] Homm F., Kaempchen N., Ota J., Burschka D. Efficient occupancy grid computation on the GPU with lidar and radar for road boundary detection. Proceedings of the 2010 IEEE Intelligent Vehicles Symposium.

[25] Tang J., Chen Y., Jaakkola A., Liu J., Hyyppä J., Hyyppä H. (2014). NAVIS-An UGV indoor positioning system using laser scan matching for large-area real-time applications. Sensors.

[26] Besl P.J., Mckay N.D. (1992). Method for registration of 3-D shapes. IEEE Trans. Pattern Anal. Mach. Intell..

[27] Censi A. An ICP variant using a point-to-line metric. Proceedings of the 2008 IEEE International Conference on Robotics and Automation.

[28] Bosse M.C. (2004). Atlas: A Framework for Large Scale Automated Mapping and Localization.

[29] Kohlbrecher S., Stryk O.V., Meyer J., Klingauf U. A flexible and scalable SLAM system with full 3D motion estimation. Proceedings of the IEEE International Symposium on Safety, Security, and Rescue Robotics.

[30] Clausen J. (1999). Branch and Bound Algorithms—Principles and Examples. Parallel Process. Lett..

